# Cloud-based applications for accessing satellite Earth observations to support malaria early warning

**DOI:** 10.1038/s41597-022-01337-y

**Published:** 2022-05-16

**Authors:** Michael C. Wimberly, Dawn M. Nekorchuk, Ramcharan R. Kankanala

**Affiliations:** Department of Geography and Environmental Sustainability, University of Oklahoma, Norman, OK 73019 USA

**Keywords:** Risk factors, Environmental impact

## Abstract

Malaria epidemics can be triggered by fluctuations in temperature and precipitation that influence vector mosquitoes and the malaria parasite. Identifying and monitoring environmental risk factors can thus provide early warning of future outbreaks. Satellite Earth observations provide relevant measurements, but obtaining these data requires substantial expertise, computational resources, and internet bandwidth. To support malaria forecasting in Ethiopia, we developed software for Retrieving Environmental Analytics for Climate and Health (REACH). REACH is a cloud-based application for accessing data on land surface temperature, spectral indices, and precipitation using the Google Earth Engine (GEE) platform. REACH can be implemented using the GEE code editor and JavaScript API, as a standalone web app, or as package with the Python API. Users provide a date range and data for 852 districts in Ethiopia are automatically summarized and downloaded as tables. REACH was successfully used in Ethiopia to support a pilot malaria early warning project in the Amhara region. The software can be extended to new locations and modified to access other environmental datasets through GEE.

## Introduction

Global environmental change has myriad influences on human health, and researchers are increasingly studying the connections between climate variation and health outcomes. Extreme heat events are becoming more frequent as the climate warms, leading to greater risk of heat-related illnesses and death^1,2^. Climate change also affects the transmission cycles of many infectious diseases and shifts the geographic distributions of important vector and host species^3^. Because climate is intimately connected with the hydrological cycle, changes in patterns of rainfall and resulting risks of flooding and drought influence the transmission of water-related diseases^4^. If the associations between environmental change and health outcomes can be elucidated, then monitoring climate variations can provide novel data on risk factors for environmentally sensitive diseases. These data can support the development of disease risk maps^5,6^, early warning systems for forecasting outbreaks of climate sensitive diseases^7,8^, and projections of the long-term effects that climate and land use change will have on human health^9,10^.

Earth-observing satellites produce an abundance of data that can be used in the study of human health. Measurements of reflected, emitted, and backscattered radiation track spatial and temporal variation in the environment, including vegetation, surface water, soil moisture, temperature, and precipitation. The resulting data provide information about habitats for vector and host species and environmental suitability for transmission of disease-causing pathogens^11–13^. Satellite remote sensing can also be used to map heat waves^14^, monitor air pollution^15^, and delineate human populations at risk of disease^16^. Most of these data are available at no cost and many have global coverage, making them particularly important for monitoring environmental health risks in low- and middle-income countries where the availability of ground-based environmental measurements is limited.

To implement climate services such as early warning systems for climate-sensitive diseases, it is essential to acquire, process, and integrate new data on a regular basis. Accessing geospatial environmental data requires navigating a vast landscape of available datasets, determining which are most suitable, and downloading large volumes of data. The data must be extracted from complex archive files, projected to new coordinate reference systems, processed to detect and fill data gaps, and summarized to generate relevant environmental indices at appropriate spatial and temporal scales^17^. Most epidemiological researchers and public health practitioners do not have expertise with satellite data and lack the necessary computational resources and specialized software. In many low- and middle-income countries, access to broadband internet is another barrier to working with large geospatial datasets^18^. There is a need for tools that facilitate the retrieval and use of satellite imagery for public health research and applications.

Cloud computing provides access to geospatial datasets along with processors, data storage, and software, as virtualized resources over the internet. Google Earth Engine (GEE) is a cloud-based system for analysis of satellite remote sensing and other gridded environmental data^19^. Computations are carried out via parallel processing in the Google Cloud, facilitating the analysis of very large datasets. A variety of data are available, including precipitation estimates, land surface temperature, and vegetation indices from Earth-observing satellites. Because data do not have to be downloaded, stored, and processed locally, GEE facilitates data access, processing, and summarization for end users with limited computational resources in low-bandwidth environments. GEE has used extensively for a variety of remote sensing applications, including public health^20^.

The goal of this study was to facilitate regular access to satellite data in support of the Epidemic Prognosis Incorporating Disease and Environmental Monitoring for Integrated Assessment (EPIDEMIA) malaria early warning project in Ethiopia^21^. To achieve this goal, we developed a GEE application called Retrieving Environmental Analytics for Climate and Health (REACH) to automate the required data processing steps and download results in a summarized format suitable for modeling malaria-environment relationships and predicting future malaria risk. We compared three different GEE implementations: 1) A data visualization and downloading tool with a menu-based user interface (UI) developed using the GEE code editor and JavaScript API, 2) An Earth Engine App that implemented this tool as a standalone web application, and 3) A Python package callable from other software to automate data processing and downloading. We assessed the strengths and limitations of each approach and considered how they can be extended to support other efforts to forecast climate-sensitive infectious diseases.

## Results

### Requirements for malaria early warning

This REACH application was developed as a component of the EPIDEMIA malaria early warning system for Ethiopia. EPIDEMIA uses routine malaria surveillance data along with multiple streams of climate data to model the effects of climate variation on malaria and generate forecasts of future malaria burden up to twelve weeks in advance. Software for data processing and harmonization, predictive modeling, accuracy assessment, and formatted report generation are implemented in the R language and environment for statistical computing^21^. Machine learning algorithms are used to model weekly fluctuations in malaria cases as a function of long-term trends, seasonal cycles, and environmental indicators related to temperature and moisture. Weekly malaria surveillance data for every district (*woreda*) in Ethiopia are collected by regional health bureaus and submitted to the Ethiopian Public Health Institute (EPHI). Daily summaries of environmental variables are also required to calibrate the malaria forecasting models and predict malaria in future weeks.

To implement EPIDEMIA, a historical database of environmental variables must first be downloaded and formatted for the initial model calibration. When EPIDEMIA is used to make forecasts, recent environmental variables are needed to generate predictions. The summarized data are expected to be tables in comma-separated value (CSV) format with one row for each combination of date and *woreda* and each environmental variable in a separate column. These files are copied into an input directory and EPIDEMIA ingests them, resolves duplicate records, fills data gaps, and links the resulting dataset to the malaria data for model calibration and forecasting. At the initialization stage, the large volume of raw historical data presents a challenge for storage and processing. When making weekly forecasts, it is essential to update the database quickly and reliably with recent observations.

Access to climate and weather data has been identified as a critical barrier to the scale-up and implementation of malaria early warning at a national level in Ethiopia. In 2020, we conducted a series of virtual workshops and interviews along with an online survey to assess the opportunities and challenges for scaling up malaria early warning in Ethiopia and documented the results in a report to the United States Agency for International Development (USAID)^22^. During workshops and interviews with participants from regional health bureaus, the Federal Ministry of Health, and academic research institutions, the problem of environmental data access was frequently highlighted as an obstacle to research and public health action related to climate and malaria. Of 22 Ethiopian malaria professionals who participated in the online survey, 12 considered environmental data access to be a major barrier to scaling up malaria early warning, 11 considered it a moderate barrier, and only one felt that it was not a barrier.

Based on this assessment and our previous experience co-developing EPIDEMIA with public health partners in the Amhara Region of Ethiopia^21^, we identified the following key requirements for climate data access to support malaria early warning.

1. Data must be freely available to users in Ethiopia and other low- and middle-income countries.
2. Variables must characterize climate-related factors that affect vector mosquitoes, including temperature, rainfall, and soil moisture availability.
3. Measurements must have high frequency with a revisit time of one week or less.
4. There must be sufficient temporal depth (15 years or more) to parameterize models based on historical malaria data.
5. Spatial extent must cover all of Ethiopia and spatial grain must be small enough to summarize individual *woredas*.
6. Data must be available within approximately one week of acquisition for use in forecasts.
7. Software must be usable on a standard computer in a regional or federal public health agency without requiring additional specialized software.
8. The interface must be efficient and processing steps must be automated so users can access data with minimal effort.
9. Data acquisition must be rapid and functional in environments with low and unstable bandwidth.
10. Outputs must be ready for automated ingestion into EPIDEMIA.

These requirements provided the guidelines for developing the data processing workflow and designing the software tools for REACH.

### Data processing workflow

**Fig. 1:**
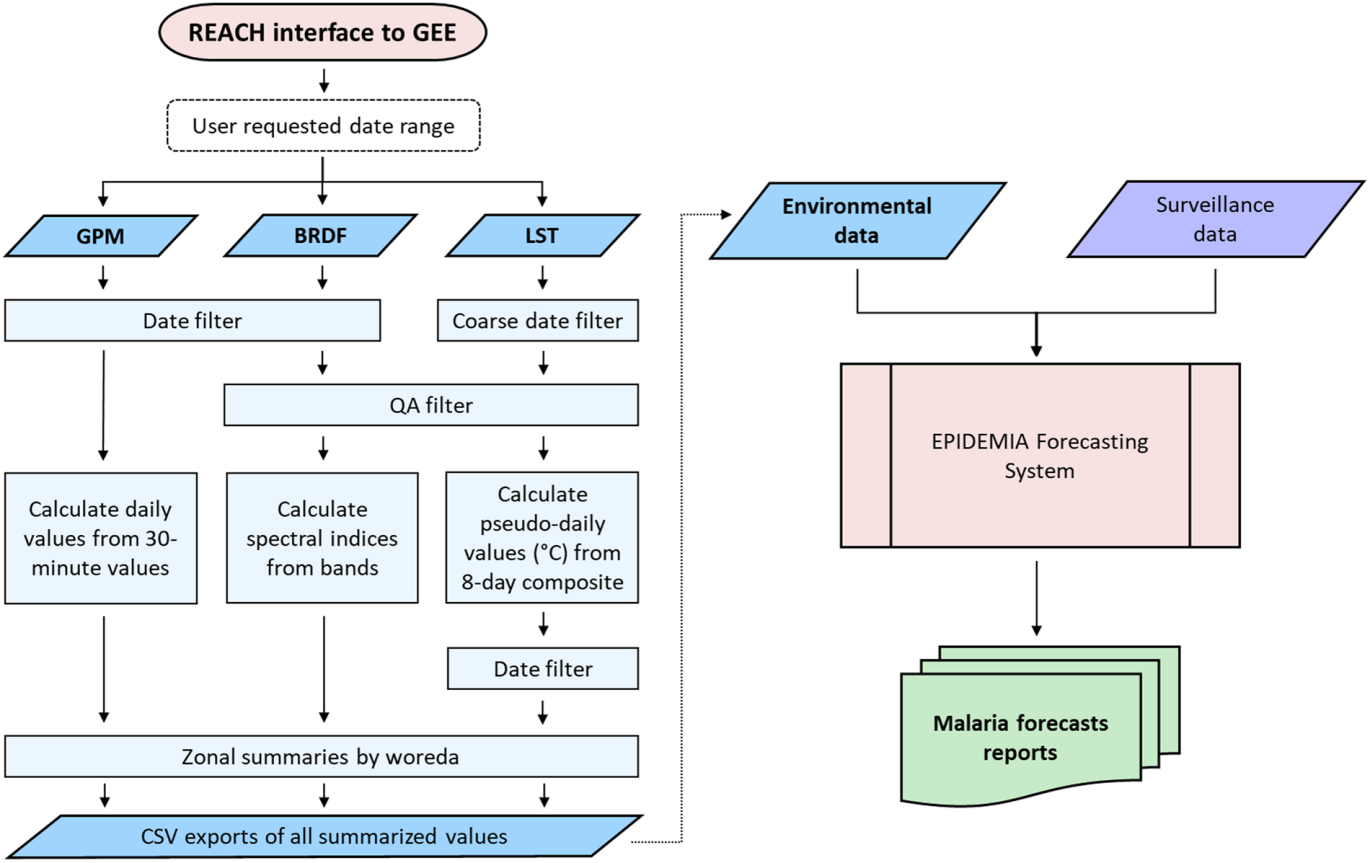
Flow diagram of REACH data processing for each of the three main sets of data: global precipitation measurement (GPM, using the IMERG6 product), spectral indices using the MODIS bidirectional reflectance distribution function (BRDF) surface reflectance product, and land surface temperature (LST) from the MODIS Terra 8-day product. The summarized output dataset from REACH feeds into the EPIDEMIA forecasting system along with epidemiological surveillance data and is used to forecast future malaria incidence.

The REACH application was designed to meet these requirements by generating daily summaries of satellite remote sensing data for every *woreda* (district) in Ethiopia (Fig. 1). These data measured environmental risk factors that have been shown to have lagged associations with malaria outbreaks in Ethiopia^23–25^. Precipitation was obtained from the Global Precipitation Mission (GPM), which produces the Integrated Multi-SatellitE Retrievals for GPM (IMERG) gridded precipitation products (Table 1). Land surface temperature (LST) and spectral reflectance from bands in the visible and infrared wavelengths were obtained from the MODerate resolution Imaging Spectroradiometer (MODIS) on board the Terra and Aqua satellites. LST variables included daytime temperature, nighttime temperature, and the mean of day and night temperatures. Spectral bands were used to calculate indices sensitive to vegetation greenness and moisture content, including the normalized difference vegetation index (NDVI), soil-adjusted vegetation index (SAVI), enhanced vegetation index (EVI), and normalized difference water index (NDWI).

**Table 1:** Remote sensing data sources and derived environmental indices.

Data Source	Indices
MODIS Terra 8-day land surface temperature and emissivity product (MOD11A2)	Daytime land surface temperature (LST), Nighttime LST, Mean LST
MODIS bidirectional reflectance distribution function (BRDF) adjusted surface reflectance product (MCD43B3)	Normalized Difference Vegetation Index (NDVI), Soil Adjusted Vegetation Index (SAVI), Enhanced Vegetation Index (EVI), Normalized difference water indices (NDWI5 and NDWI6)
Integrated Multi-SatellitE Retrievals for GPM (IMERG) product version 6	Total precipitation

**Fig. 2:**
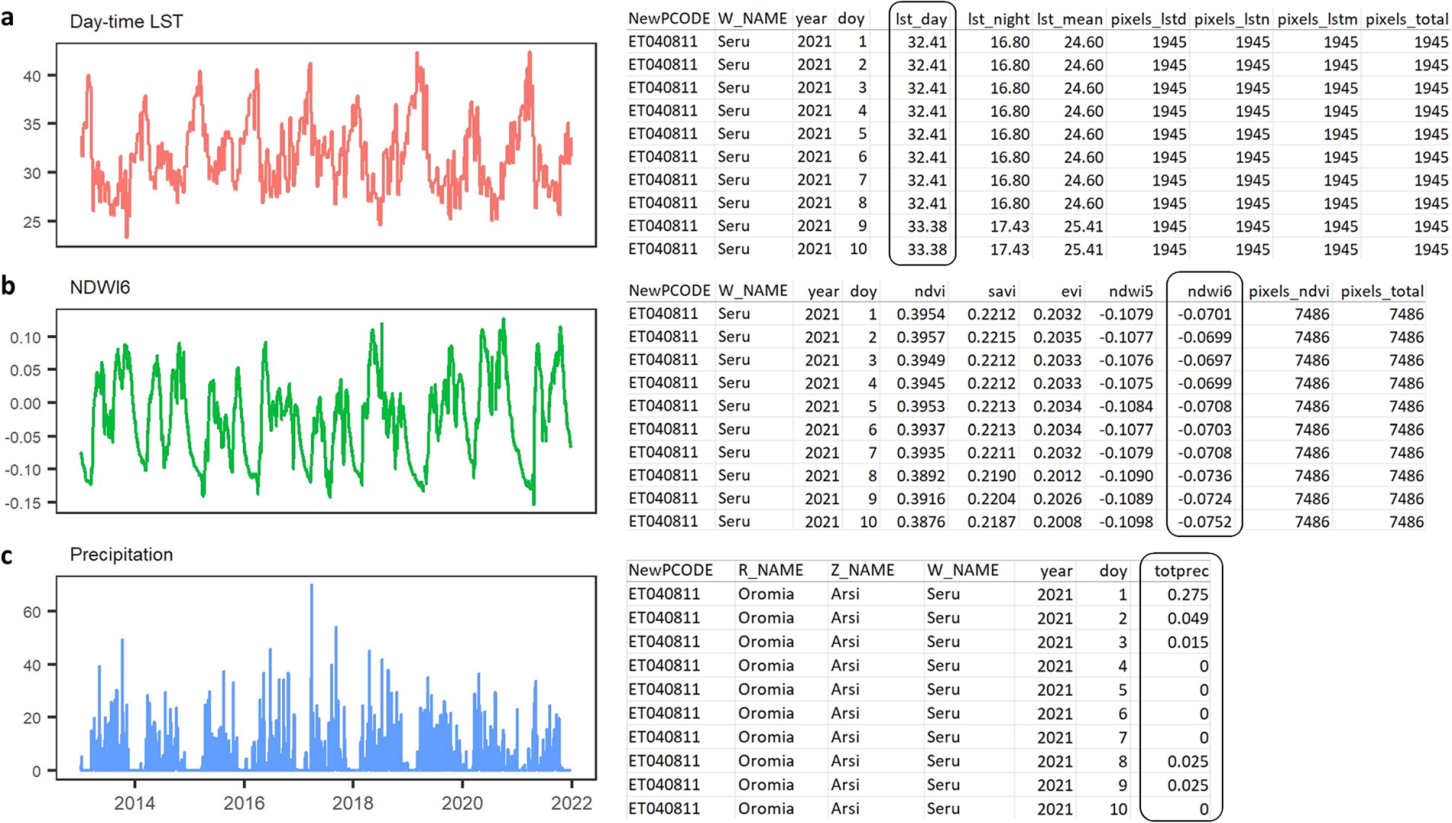
Example time series plots and CSV output screenshots for the *woreda* Seru in the Arsi zone of the Oromia region. The plot shows three environmental variables: (**a**) daytime land surface temperature (the “lst_day” column in the LST CSV file), (**b**) NDWI6 (the “ndwi6” column from the spectral indices CSV file), and (**c**) precipitation (the “totprec” column from the precipitation CSV file) for 2013 through 2021. The LST and spectral indices files also contain information about the numbers of cloud-free pixels and total pixels in the *woreda*. The first ten days of 2021 are shown in the CSV outputs.

To run the REACH application, the only input required from the user was the range of dates over which to process and download data. The software then queried the GEE cloud-based data archive to obtain gridded remote sensing data falling within the selected date range and the geographic boundaries of Ethiopia. Cloudy pixels and other low-quality observations were screened out of the MODIS data using the pixel-level quality assurance (QA) data layers for each MODIS product. Next, these datasets were harmonized over time to have matching daily resolutions and daily environmental indices were computed for each grid cell. The *woreda* boundaries were overlaid on the environmental indices and daily zonal means were computed for every *woreda* (Fig. 2).

The summarized data were formatted as tables where each row was referenced by a unique combination of date, year, *woreda* name, and region and zone names. For the MODIS variables, the tables included the total count of grid cells and the number grid cells containing valid data. These tables were downloaded as CSV text files. This process greatly reduced the size of downloads and made it feasible to use the application in Ethiopia where internet connections are often slow and unstable. For example, the size of the raw data files required to compute a year of daily environmental indices for all of Ethiopia was approximately 3.8 TB, whereas the size of the CSV file with one year of daily *woreda*-level summaries was only 88 MB. The volume of data that had to be downloaded and stored was reduced by a factor of more than 43,000. Because all computation took place in the cloud, it was not interrupted by temporary slowdown or loss of internet connectivity during the data processing stage. Using this approach, we generated an 20-year archive of historical *woreda*-level data summaries from 2002-2021^26^.

### JavaScript API implementation

**Fig. 3:**
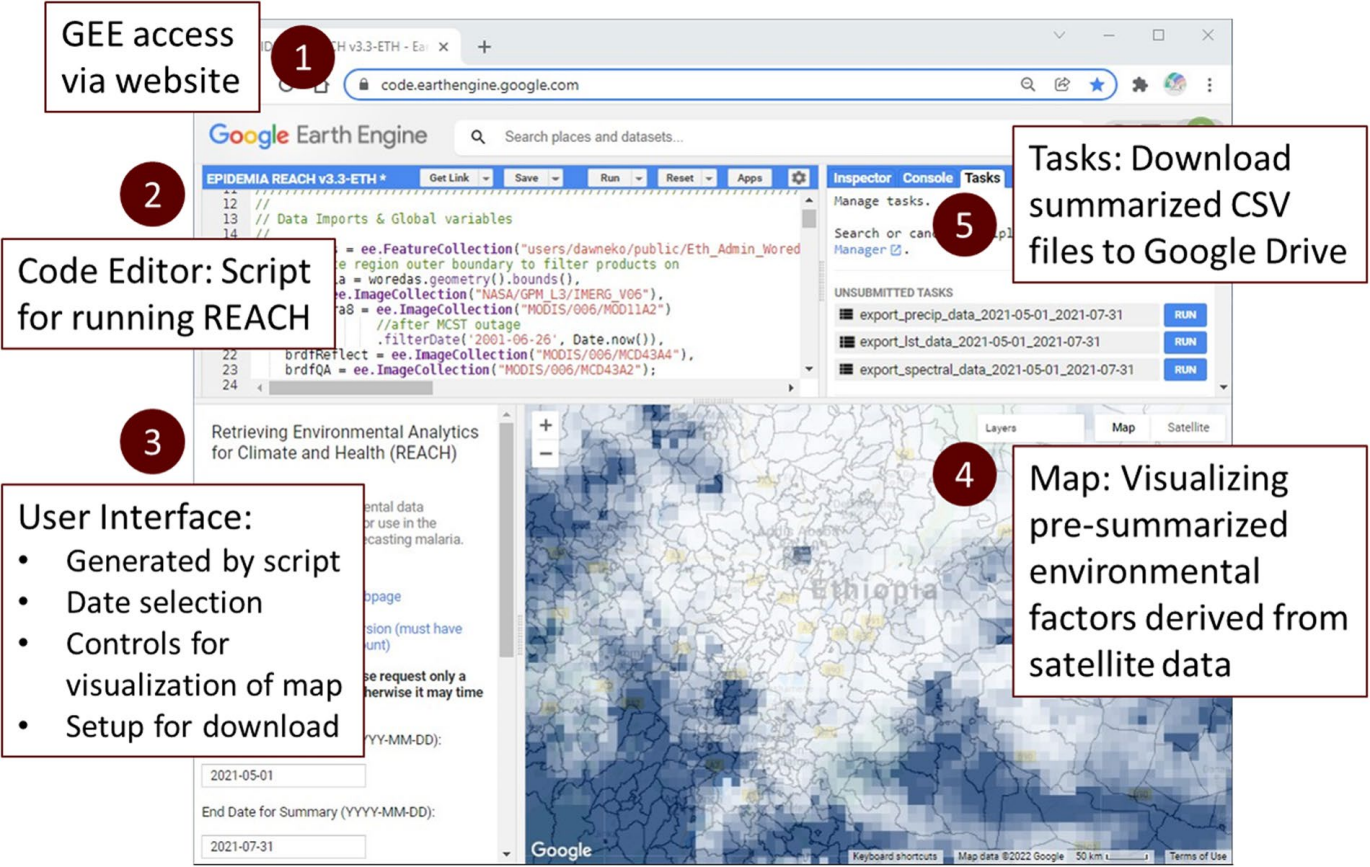
Screenshot of REACH UI in the Google Earth Engine (GEE) Code Editor with descriptions of the sections. 1) GEE is accessible in a web browser via a universal resource locator (URL). 2) The Code Editor pane contains the script that is ‘Run’ using the button on the header. 3) The user interface is created by the script when it is run, and this is where the user inputs the start and end dates for the download. 4) The map window provides visualization of the environmental variables and woreda boundaries. 5) The summaries for the selected date range are sent to the Tasks pane, where the user can initiate processes for the CSV files to be generated in the cloud and downloaded to the user’s Google Drive.

We initially implemented REACH using the GEE code editor and the JavaScript API (Fig. 3). The code editor provides a web-based interactive development environment (IDE). The JavaScript API includes a variety of functions for accessing and processing remote sensing in the cloud and a user-interface (UI) package for creating user controls and displaying maps and charts. Downloads to Google Drive can be initiated by a script and are monitored via the task manager window in the code editor. To access the code editor, the user must have a Google account and email along with an associated GEE account, which is available upon request from Google. The REACH script can be accessed by pasting the text into the code window (Fig. 3 Pane 2), saving and opening scripts from the user’s account in the script manager window, or accessing a shared script from another GEE account via a URL.

**Fig. 4:**
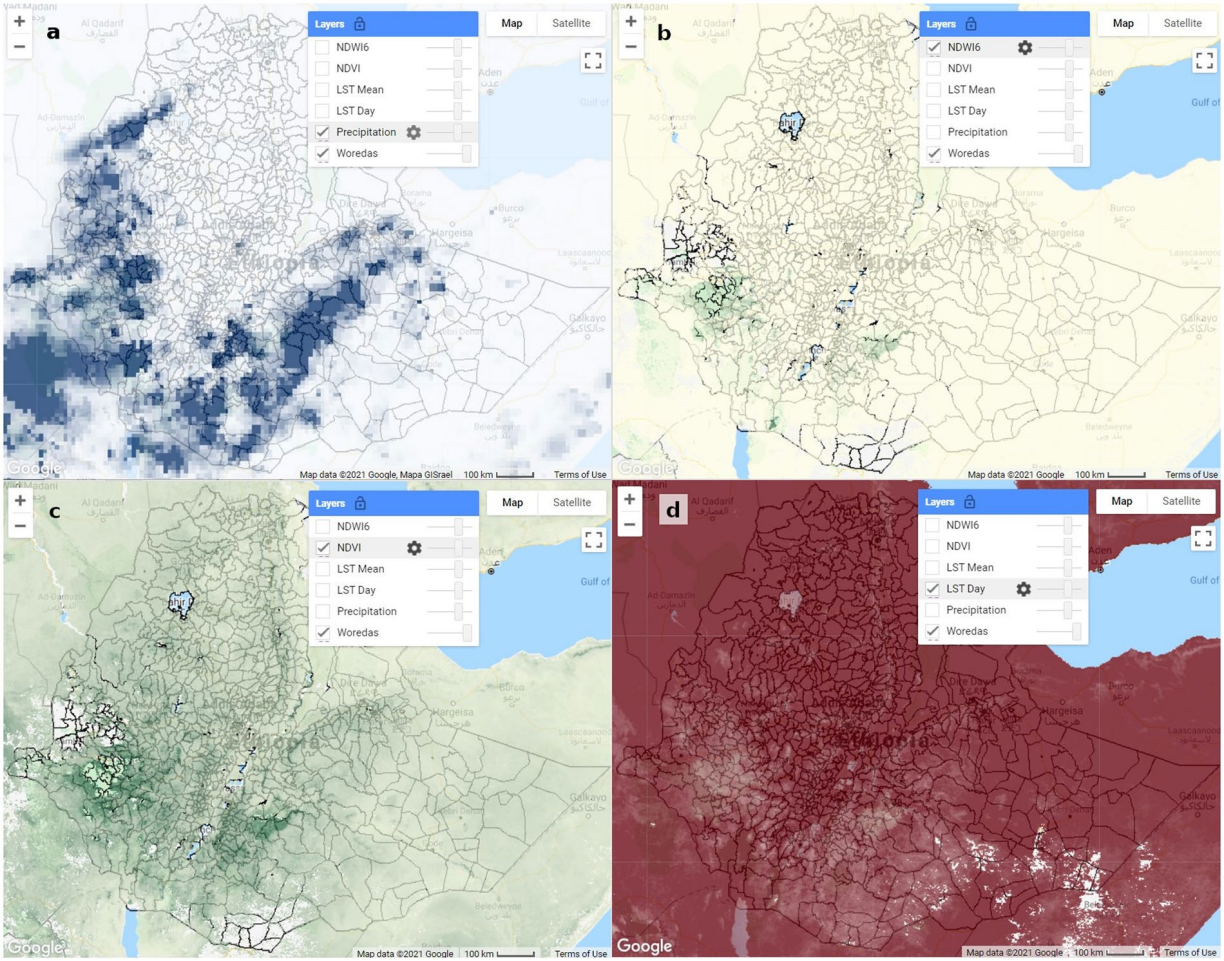
Visualizations from the REACH map window showing examples of remote sensing data with *woreda* boundaries: (**a**) precipitation, (**b**) NDWI6, (**c**) NDVI, and (**d**) daytime LST (environmental data shown for 18 March 2021).

Once the script is loaded, clicking the run button initiates the script and loads the UI (Fig. 3 Pane 3). The user specifies a range of dates and the script downloads and processes the remote sensing data for that period. The data can then be downloaded by initiating and running a set of GEE tasks that write the output to the user’s Google Drive (Fig. 3 Pane 5). The user can also visualize the data by choosing a date, selecting which environmental indices to display, and panning and zooming in the map interface (Fig. 3 Pane 4 and Fig. 4). The user interface was purposefully designed to be simple to facilitate routine data access with a few clicks as possible while also provide some basic capabilities for data visualization and exploration.

### Earth engine app

**Fig. 5:**
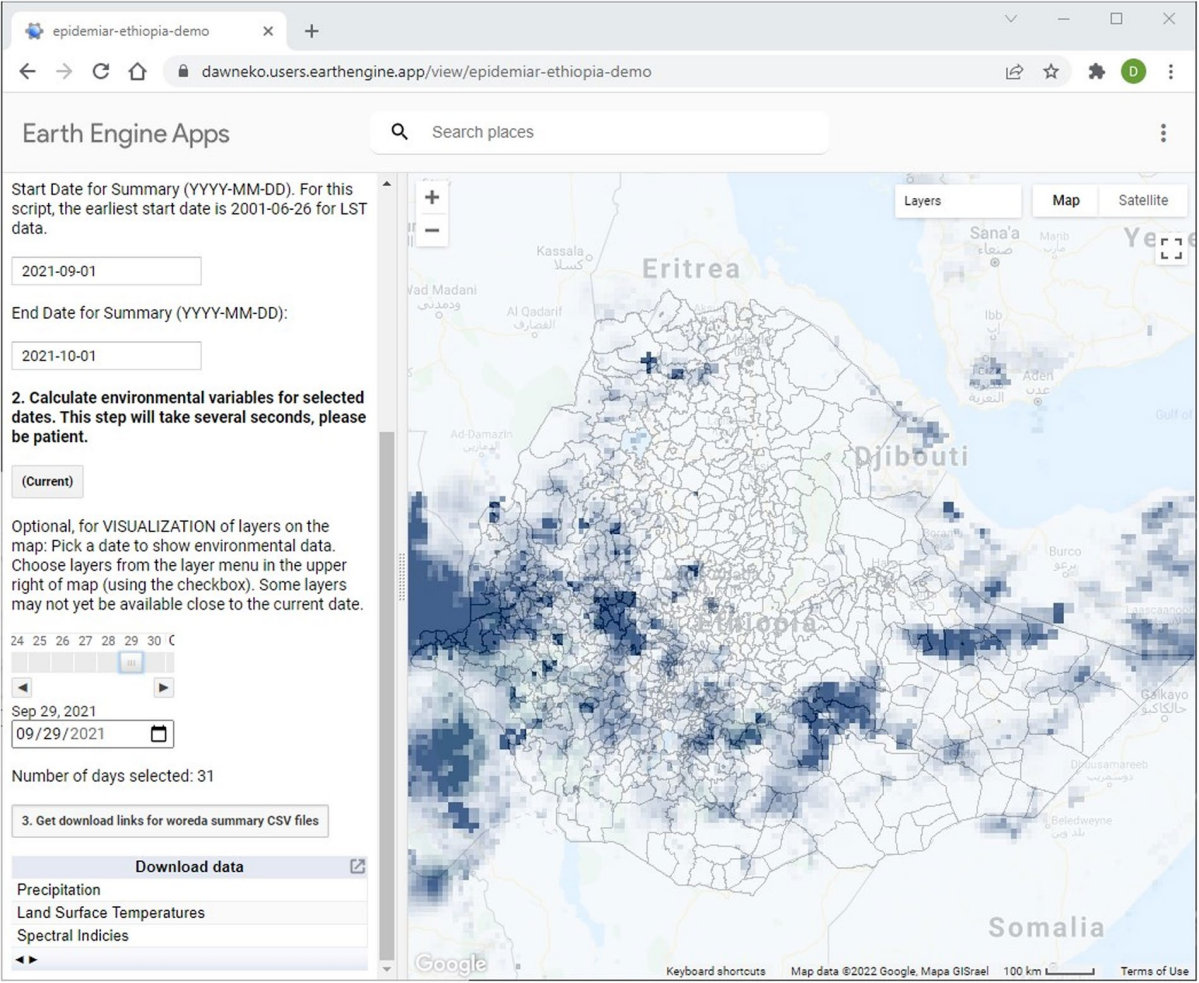
Screenshot of the publicly accessible REACH Earth Engine App (accessible at https://dawneko.users.earthengine.app/view/epidemiar-ethiopia-demo).

The REACH script was also implemented as a dynamic, publicly accessible web application (Fig. 5). GEE provides tools that allow scripts developed in the code editor to be published as GEE apps. The user interface is essentially the same as when the script is run in the code editor, although the UI must be designed to operate without access to the code, console, or tasks panes. However, the app can be accessed directly from a public URL and does not require a GEE account to run. The downloads are limited to shorter time ranges than those made through the JavaScript API, but they are downloaded directly from the browser rather than being added to the user’s Google Drive. For users with GEE accounts that need access to the JavaScript API version, a link is provided that brings the user to the same code base in the GEE code editor browser page.

### Python API implementation

Both the GEE Code Editor and the Earth Engine App require manual inputs from the user to initiate the software, specify dates of interest, and download the data. The Python API implementation allows for all these steps to be controlled from a Python script run on the client workstation. The REACH application was implemented as a Python package with a function called gee_to_drive() with two arguments for start date and end date. After installing the package, the user must authenticate their GEE account and save an authentication token to the local workstation. The gee_to_drive() function can be called by Python script, or called from the R environment using the reticulate package. The script initiates data acquisition and processing in GEE and results are downloaded to the user’s Google Drive account. Using this approach, remote sensing data acquisition via GEE can be incorporated directly into software applications that also ingest the data, harmonize them with other datasets, apply predictive models, and generate forecasting reports. The gee_to_drive() function can also be used with job scheduling software to automatically update remote sensing data on a regular basis.

## Discussion

The REACH application was successfully used in the pilot implementation of the EPIDEMIA malaria early warning system in the Amhara region of Ethiopia during 2019-2020. In an earlier version of EPIDEMIA, we used a client-side application that automatically downloaded remote sensing data and conducted all necessary data processing and summarization on a client-side computer^17,21^. This approach was successful when implementing EPIDEMIA on computers at a university in the United States. However, it was not feasible for transferring the technology to partners in Ethiopia because internet connectivity was a barrier to data access and the available computer resources did not provide enough data storage and processing capabilities. The REACH application for GEE was developed to address these issues and provide a software tool for environmental data access that could be implemented by our partners in the public health sector.

**Fig. 6:**
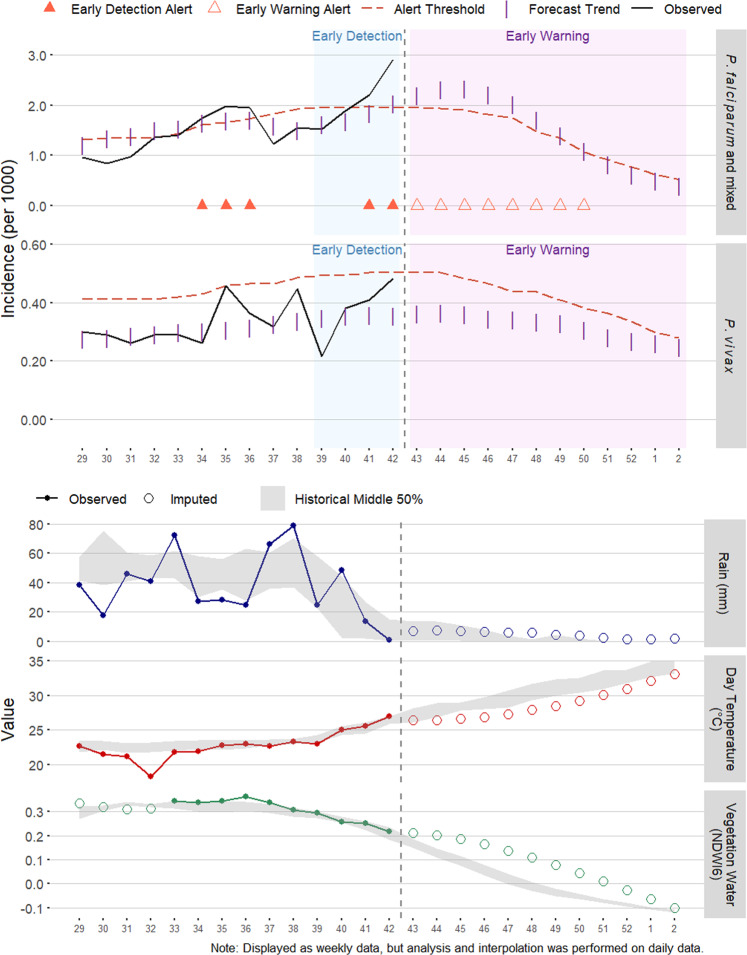
Example *woreda*-level timeseries charts from an EPIDEMIA forecast report for 2019 week 42. The top two series show the observed malaria incidence (solid lines) up to week 42 and forecasted incidence (vertical bars) through week 2 2020 (12 weeks ahead) along with expected malaria incidence (dashed lines) with alerts for early detection and early warning of outbreaks (triangles). The bottom three timeseries show the observed environmental variables from REACH (solid lines) with historical interquartile ranges (gray envelopes) and future extrapolations (circles).

**Fig. 7:**
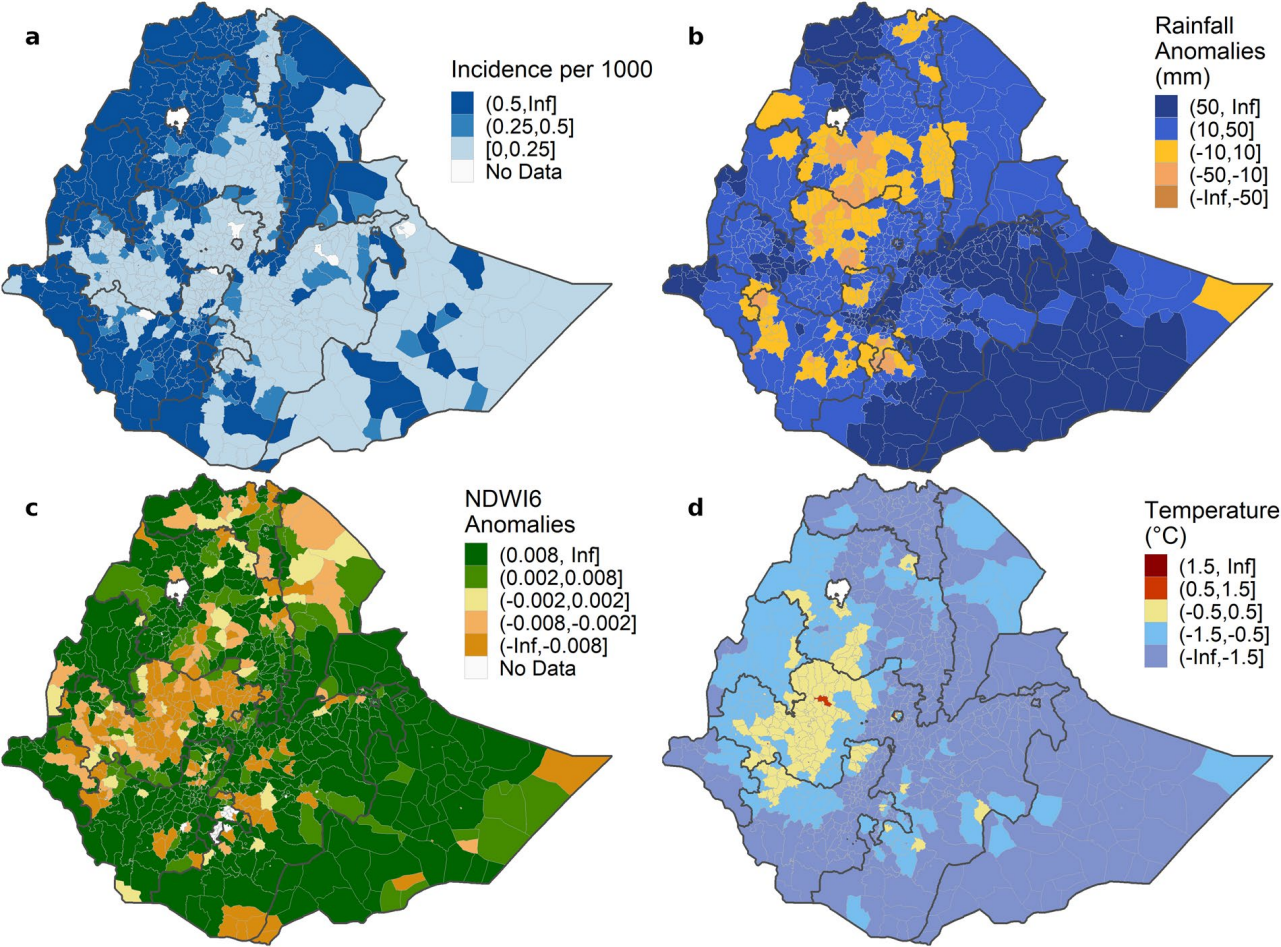
Example maps showing (**a**) *P. falciparum* malaria incidence, climate anomalies for (**b**) rainfall, (**c**) NDWI6, and (**d**) daytime LST for a four-week time period of week 39 through 42 2019. Incidence is reported per 1000 population, and anomalies are defined as the departure of the observed values from historical climate averages from 2002 to present.

Users working at Bahir Dar University and the Amhara Regional Health Bureau were able to use REACH through the GEE code editor and JavaScript API to download data on a weekly basis and incorporate them into the EPIDEMIA system for predicting future malaria risk. An important goal of the pilot implementation was to allow the users to obtain data and generate a forecast in no more than one hour, and this goal was achieved. Although unstable internet connectivity presented a challenge, the relatively simple user interface and the small sizes of the downloaded data summaries resulted in reliable data access. In EPIDEMIA, the data accessed with REACH were harmonized with malaria surveillance data and incorporated into routine reports. District-level charts displayed time series of historical climate and malaria patterns and future forecasts (Fig. 6). Maps of malaria incidence and environmental variables helped to visualize locations with high malaria burden and unusual climate anomalies (Fig. 7).

We examined three different approaches to implementing REACH in GEE, and each had strengths and limitations. Running the JavaScript API implementation of REACH through the GEE code editor is relatively simple after initial implementation but still allows considerable flexibility. For example, it is straightforward to upload a new dataset of updated *woreda* boundaries if they need to be changed, and the code can be modified to use REACH in new locations or add new data sources to the summaries. However, the requirement to have a GEE account and the need to navigate the code editor interface could be barriers for some users. Although the interface was designed to minimize the actions require for data access, additional steps are required to log into Google, load and run the script, and start the downloads via the Tasks tab. Also, modifying the code base requires programming experience combined with knowledge of the GEE JavaScript API.

The GEE App version can be made publicly accessible and run from a URL without logging in to a Google account. This simplicity is an advantage for operational malaria early warning because fewer steps are required to download the new data required to make a forecast. However, our experience has shown that large data requests encompassing multiple years often result in timeouts and other errors when processed through the app. Thus, the app is best suited for quickly downloading several weeks or months of data. Requests that require longer processing time, for example gathering many years of historical data, still need to be done using the JavaScript or Python API. With the appropriate UI design and coding, the same script can be run in the code editor and used to create an app.

Running REACH through the GEE Python API offers the prospect of automating remote sensing data access and incorporating it directly into software for malaria early warning. The Python API can be accessed directly from a client computer and does not require manual inputs through the GEE code editor. Because EPIDEMIA is implemented in R, the reticulate package can be used to call Python from R and a scheduling package like taskscheduleR can be used to automate the downloads. However, automation makes the system more complex and may ultimately be more challenging to manage than data access through an external application that requires some manual steps. For implementation in public health agencies in Ethiopia, we determined that using the browser-based GEE App, which required a few manual steps to enter dates and download summary files, was the most sustainable approach for enabling routine access to district-level climate data.

Missing data caused by clouds are a common issue with optical and thermal remote sensing, particularly in tropical climates that have an extended rainy season^27^. In the REACH application, cloudy pixels were a common occurrence in the MODIS data, particularly from June-August. We removed these data values, computed the daily zonal statistics for each *woreda* using only valid pixels, and reported the number of cloud-free pixels used to calculate each summary statistic (Fig. 2). Where there were no cloud-free pixels within a *woreda*, no value was returned. When the *woreda*-level summaries are incorporated into EPIDEMIA, an algorithm is applied to screen missing and low-quality observations and to identify and impute missing values. An alternative approach would be to impute missing data at the pixel level while processing the data in GEE. However, this approach would necessitate a more complicated set of scripts and require more computation time.

The methods used in the REACH application can be readily expanded to new locations and different types of geospatial environmental data. The approach can also be extended to different cloud platforms. We implemented REACH in Google Earth Engine because it is the most matured cloud-based application currently available for accessing and processing satellite data. Other commercial cloud computing service providers, including as Microsoft Azure and Amazon Web Services, also provide access to extensive satellite data archives via their platforms. The results of our pilot implementation emphasize that the cloud-based approach is feasible and highly effective at providing rapid and timely access to climate data to support climate-sensitive disease forecasting at regional and national levels.

## Methods

The REACH application used three remote sensing datasets (Table 1). Land surface temperature (LST) data were from the MODIS Terra 8-day product (MOD11A2). Spectral indices were calculated using the MODIS bidirectional reflectance distribution function (BRDF) adjusted surface reflectance product (MCD43B3). Rainfall data were obtained from the Integrated Multi-SatellitE Retrievals for GPM (IMERG) product version 6, where GPM is an acronym for the Global Precipitation Measurement mission. Details of the processing of each data source are provided below.

### Land surface temperature

We extracted day and night LST values for every 1000 m pixel within Ethiopia along with associated quality assurance (QA) fields. We labelled all pixels that had quality assurance (QA) values greater than or equal to two in either daytime or nighttime images as missing data. The MOD11A2 LST product is provided as 8-day means, which were converted to daily values by assuming a constant temperature during each 8-day composite period. Mean daily temperature was the mean of day and night observations. LST measures the temperature of the uppermost surface of the Earth, which may consist of soil, vegetation, impervious surfaces, or other types of land cover depending on the location. LST is often used as a proxy for near-surface air temperature in situations where ground-based meteorological measurements are not available. The relationship between LST and air temperature is complex and depends on land cover, time of day, and local meteorological conditions. However, spatial and temporal variation in LST and near-surface air temperature are often strongly correlated^28,29^.

### Spectral indices

The MCD43B3 data provide daily surface reflectance data in the visible, near infrared, and shortwave infrared spectra at a 500 m resolution along with QA fields. We labelled all pixels that had QA values from the NIR band greater than or equal to two as missing data and masked out permanent water bodies. The surface reflectance bands were used to compute a set of spectral indices. The Normalized Difference Vegetation Index (NDVI), Enhanced Vegetation Index (EVI), and Soil-Adjusted Vegetation Index (SAVI) measure green vegetation^30–32^. Although vegetation greenness generally does not have a direct influence on malaria transmission, seasonal and interannual variation in greenness can be a sensitive indicator of environmental factors such as temperature and rainfall^33^. The Normalized Difference Water Index (NDWI) measures the amount of liquid water in vegetation and is sensitive to variation in soil moisture^34^. Two versions of the NWDI were calculated using two different shortwave infrared bands, the 1230–1250 nm MODIS band five (NDWI5) and the 1628–1652 nm MODIS band six (NDWI6).

### Rainfall

The suite of IMERG products provide global precipitation estimates from 2000 to present using data from the current Global Precipitation Measurement (GPM) mission, the older Tropical Rainfall Measurement Mission (TRMM), and other sources^35^. Three IMERG products provide precipitation estimates at 30-minute intervals for 0.1 arc degree pixels (approximately 11.1 km at the equator). The Early Run is an initial estimate produced with a minimum latency of four hours, the Late Run provides an updated estimate based on more data with a minimum latency of twelve hours, and the Final Run incorporates monthly rain gauge data with a latency of several months. The GEE IMERG data asset combines all these products. Initially, the Early and Late Run products are ingested and then replaced with the Final Run data when they are available.

### Zonal statistics

All variables were summarized as zonal means by *woreda*. These are the smallest administrative units in Ethiopia for which weekly surveillance data are routinely compiled and are thus the level at which climate-malaria associations are analyzed and predictive models are developed. Geospatial data delineating the boundaries of administrative units, including *woredas*, zones, and regions, are produced by the Ethiopian Mapping Agency. We obtained a dataset of 2019 administrative boundaries within Ethiopia from USAID/Ethiopia. The boundaries of many *woredas* have changed over time as they have been have subdivided or undergone other reconfigurations. We therefore used multiple sources of historical data to construct a set of harmonized *woredas* that could be associated with long-term malaria surveillance data collected across changing district boundaries. These harmonized woredas were uploaded to GEE as a publicly accessible asset and used to summarize the environmental data.

Daily variables of the land surface temperature variables, precipitation, and spectral indices were summarized by taking the mean of all available values within each *woreda*. The IMERG precipitation data were resampled to a 1000 m resolution to ensure that there was at least one grid cell within each *woreda* for the zonal summaries. For the MODIS LST variables and spectral indices, the total number of cloud-free pixels used to generate the zonal summaries was reported along with the total number of pixels in the *woreda*. Where there were no cloud-free pixels within a *woreda*, no value was returned.

## Data Availability

All of the raw data used by REACH are from publicly available GEE datasets that can be accessed by any user with an account. https://developers.google.com/earth-engine/datasets. Summarized data for Ethiopia can be downloaded directly using the REACH app, which can be accessed using the following URL: https://dawneko.users.earthengine.app/view/epidemiar-ethiopia-demo. An archive of daily environmental indices for all woredas in Ethiopia from 2002–2021 was generated using REACH and archived on figshare^26^.

## Code availability

The REACH software, including JavaScript code, a sample R script for running REACH using the Python API, and a user guide, is accessible via the following GitHub archive: https://github.com/EcoGRAPH/epidemia_reach. Computer code for the REACH Python package is accessible via the following GitHub archive: https://github.com/EcoGRAPH/epidemia_gee.
